# COVID-19 in early 2021: current status and looking forward

**DOI:** 10.1038/s41392-021-00527-1

**Published:** 2021-03-08

**Authors:** Chengdi Wang, Zhoufeng Wang, Guangyu Wang, Johnson Yiu-Nam Lau, Kang Zhang, Weimin Li

**Affiliations:** 1 Department of Respiratory and Critical Care Medicine, National Clinical Research Center for Geriatrics, Frontiers Science Center for Disease-related Molecular Network, West China Hospital, West China Medical School, Sichuan University, Chengdu, China; 2 School of Information and Communication Engineering, Beijing University of Posts and Telecommunications, Beijing, China; 3 Department of Applied Biology and Chemical Technology, Hong Kong Polytechnic University, Hong Kong, China; 4 Center for Biomedicine and Innovations, Faculty of Medicine, Macau University of Science and Technology, and University Hospital, Macau, China

**Keywords:** Respiratory tract diseases, Infectious diseases

## Abstract

Since the first description of a coronavirus-related pneumonia outbreak in December 2019, the virus SARS-CoV-2 that causes the infection/disease (COVID-19) has evolved into a pandemic, and as of today, >100 million people globally in over 210 countries have been confirmed to have been infected and two million people have died of COVID-19. This brief review summarized what we have hitherto learned in the following areas: epidemiology, virology, and pathogenesis, diagnosis, use of artificial intelligence in assisting diagnosis, treatment, and vaccine development. As there are a number of parallel developments in each of these areas and some of the development and deployment were at unprecedented speed, we also provided some specific dates for certain development and milestones so that the readers can appreciate the timing of some of these critical events. Of note is the fact that there are diagnostics, antiviral drugs, and vaccines developed and approved by a regulatory within 1 year after the virus was discovered. As a number of developments were conducted in parallel, we also provided the specific dates of a number of critical events so that readers can appreciate the evolution of these research data and our understanding. The world is working together to combat this pandemic. This review also highlights the research and development directions in these areas that will evolve rapidly in the near future.

## Introduction

In December 2019, a novel pneumonia with a high potential of transmissibility between humans was first reported. As a number of the initially identified cases had visited a large seafood and live animal market, some investigators were prompted to have an unconfirmed suspicion that this might be the initial source of infection.^1,2^ The Chinese Center for Disease Control and Prevention, along with other related institutions, quickly identified the pathogen as a new type of coronavirus. To ensure that the information is shared quickly across the world, the first viral sequence was deposited into GenBank and made public on 26 December 2019 (LR757995, LR757998).

World Health Organization (WHO) issued alerts on 30 December 2019 and on 30 January 2020, and declared this viral infection as a Public Health Emergency of International Concern. On 11 February 2020, the International Committee of Taxonomy of Viruses named this virus Severe Acute Respiratory Syndrome (SARS)-CoV-2 based on the phylogenetic relationship of the coronavirus that caused the SARS outbreak in 2003. On the same day, WHO announced COVID-19 as the name of this novel disease caused by this virus following the guidelines of the World Organization for Animal Health and the Food and Agricultural Organization of the United Nations.

As of 1 February 2021, 13–14 months after the first description of the virus, there are >100 million subjects globally (from more than 210 countries) with confirmed SARS-CoV-2 infection based on the molecular assay. More than 2 million deaths have been attributed to COVID-19.^3^ This pandemic has posed a great menace to human physical and mental health and has dramatically impacted the daily life with psychosocial implications on a global scale. This brief review (with updates up to 9 February 2021) summarized our knowledge and advanced on epidemiology, virology and pathogenesis, disease diagnosis, use of artificial intelligence in assisting diagnosis, treatment, and vaccine development.

## Epidemiology of SARS-CoV-2 infection and public health impact

At the initial phase of the COVID-19 outbreak, the linkage between newly identified patients and their recent visits to the Seafood Wholesale Market suggested a potentially zoonotic origin of the virus.^1,2^ Although the original and intermediate animal hosts for SARS-CoV-2 have not yet been definitively determined, the phylogenetic proximity of the SARS-CoV-2 to the coronaviruses from bats suggested the possibility that this novel virus may be related to the coronavirus from bats.

In January 2020, there was strong clinical evidence confirming the human-to-human transmission of SARS-CoV-2. The relative high infectivity, upper respiratory mode of transmission (may also be an element of transmission by contact), the relatively long incubation period, and the long viral shedding period, together with the current global travel pattern, constitute all the key elements for this virus to evolve into a pandemic quickly.^1,2,4,5^

Current evidence has indicated that SARS-CoV-2 could be transmitted through various routes. This is not surprising as the viral receptor is human angiotensin-converting enzyme 2 (hACE2), which is expressed in a wide range of cell types, including lung alveolar cells, endothelial cells, blood vessels, gastrointestinal, and liver cells. As *hACE2* is an essential gene, the entire human population is susceptible to SAR-CoV-2. There is as yet no publication that any genetic polymorphism of hACE2 is associated with resistance to SARS-CoV-2 infection. At present, a number of studies confirmed the transmission of SARS-CoV-2 through respiratory droplet transmission.^6,7^ There is also strong evidence that physical contact with infected subjects or contaminated items can transmit this virus.^8^ Healthcare workers taking care of screening for COVID-19 subjects and family members of COVID-19-infected subjects are at high risk of being infected.^9^ As the gastrointestinal tract is also an infected organ, and there are reports of SARS-CoV-2 detected in feces,^10^ it is possible that fecal–oral transmission can occur. There is also a report showing that SARS-CoV-2 can infect conjunctiva cells, suggesting that this can be another route of transmission.^11^ Whether maternal–fetal transmission can occur remains to be established.^12^

The public health impact of the SARS-CoV-2 pandemic is beyond everybody’s imagination. This pandemic has affected more than 210 countries and a majority of these countries are still under some infection control measures, including quarantine, lockdown, and recommended or mandatory general facemask use, and social distancing in public areas. As of 1 February 2021, >100 million people have been confirmed to have SARS-CoV-2 infection based on molecular assays that detect the viral nucleic acids (i.e., the virus). If one considers the number of subjects who were relatively asymptomatic or with mild symptoms and those that might not be tested for various reasons, the total number of subjects infected by SARS-CoV-2 is likely to be much >100 million. It is also important to note that >2 million have already died from SARS-CoV-2 infection (i.e., COVID-19).

A number of publications have described mathematical models trying to report and project the epidemiology of this infection.^10,13–16^ Experiences were mainly based on data from the early affected countries. We have also established a four-compartment model and took into consideration both social interaction factors and viral transmissibility factors.^16^ When the model was applied to data from Italy, UK, and the USA, it was estimated that the infection likely started in these places at around the same time, which is not surprising given the high level of travelers among epidemic regions. The model was able to estimate the impact of various public policies on the cumulative number of infected cases. The sad news is that this model predicted that with the current effectiveness of the policies (not extremely effective), SARS-CoV-2 infection, without an effective drug treatment or an effective vaccine for general use, is likely to stay for quite a while, even becoming seasonal. Given the need for various governments to reopen their countries to balance the spread of their infection vs the psychosocial, mental, and economic impact, many strategies were also proposed including staged reopening with intensive monitoring of new cases.^16,17^ Our model showed that this measure may work if there are significant social distancing and partial lockdown in conjunction with general facemask use, and intensive monitoring for new cases to avoid a relockdown.^16^ When we reviewed the various policies implemented in different countries, the compliance of the local population in the past 12 months, and the country’s infection rate, most of the outcome were in line with the predictions generated by the mathematical model.

## Clinical features

The initial clinical symptoms of COVID-19 are similar to all types of viral pneumonia, with varying degrees of severity. The incubation period of SARS-CoV-2 is generally between 3 and 7 days [US Center of Disease Control (CDC) estimated a 2–14 day range], with the shortest being 1 day and the vast majority within 2 weeks. A proportion of infected subjects may remain asymptomatic. Fever, cough, and shortness of breath were the first typical symptoms of COVID-19 pneumonia initially highlighted by CDC, and chills, muscle pain, sore throat, and new loss of taste of smell were later added to the list.^18^ Some patients have headache and myalgia, and others might have diarrhea, suggesting the involvement of the gastrointestinal tract. Patients with severe symptoms usually experience chest tightness and dyspnea in ~7–10 days after the onset of symptoms, and a proportion will progress to develop acute respiratory distress syndrome, septic shock, metabolic acidosis, and coagulopathy. It is also worth noting that some severely ill patients initially have mild symptoms like low-grade fever and mild cough, but rapidly deteriorate.^19^ The pathophysiology involved with this rapid progress in this subset of patients remains to be determined.

Among the subjects showing symptoms (the COVID-19 disease), ~80% of patients had a mild illness, 14% of patients showed severe illness, and 5% of patients developed critical illness requiring intensive care or mechanical ventilation assistance.^20^ Elderly people and people with comorbidities such as chronic obstructive pulmonary disease, diabetes, hypertension, and heart disease have an increased risk of severe illness. Importantly, some of these patients also had mild symptoms initially and progressed rapidly later.^19^ There was a suggestion that patients on ACE inhibitors are prone to have more severe disease due to the induction of a higher level of hACE2 expression, but this has not been confirmed.

From a clinical management perspective, it is challenging to correct hypoxia with mechanical ventilation in critically ill patients. Some clinicians suggested the possibility of pulmonary vasculature involvement leading to a mismatch of the ventilation/perfusion system as both the ventilation and perfusion were affected in the pathogenesis. This line of research is certainly worth pursuing as the understanding of the pathogenesis based on the clinical hints may improve the precision of clinical management strategies.^21^

Based on the latest information, most COVID-19 patients recovered, while a small subset of patients (from 0.5 to 5% depending on their access to proper treatment and preinfection health conditions) with severe illness will have severe/critical illness.^22^

We have published the observation that in some recovered patients, the antibody titer dropped very quickly, which suggested that they may be susceptible to reinfection by SARS-CoV-2 again.^23^ Along the same line, we described two patients in a cohort of 193 recovered patients who were diagnosed to have SARS-CoV-2 reinfection within 3 months of their first infection.^24^ This observation was later observed by other investigators.^25^ Reinfections hint that immunity against COVID-19 may be fragile and wane relatively quickly, with implications not just for the risks facing recovered patients, but also for how long future vaccines might protect people. There will certainly be more clinical data in the direction available in the near future.

### Latest clinical data from China

As of 1 February 2021, among the 101,039 cases in China diagnosed with COVID-19, 93,726 patients recovered and 4826 patients died, with an overall case fatality rate of 4.8%.^3^

The Chinese Center for Disease Control and Prevention published the epidemiological data of COVID-19 in China based on an earlier dataset.^20^ Of the 72,314 “suspected” COVID-19 cases reported in mainland China, 44,672 (61.8%) were diagnosed to have COVID-19. Among the “suspected” and confirmed COVID-19 subjects, only 889 (1.2%) were asymptomatic. It is important to note that the denominator of this survey was based on suspected cases, that is, those with symptoms or closed contacts, and therefore, the total percentage of asymptomatic cases in reality can be much higher. The majority of the confirmed patients was between 30 and 79 years old (86.6%), and ~51% of these patients are male. In this cohort, there were 1023 deaths among the confirmed cases with a crude mortality rate of 2.3%, a male mortality rate of 2.8%, and a female mortality rate of 1.7%. The crude mortality rate in the ≥80-year-old age patients was 14.8%, and the critical case mortality rate in critical cases was 49%. The crude mortality rate of patients with unreported comorbidities was ~0.9%, and the mortality rate of patients with comorbidities was much higher: 10.5% for patients with cardiovascular diseases (with no information yet on whether these patients were ACE inhibitor takers), 7.3% for patients with diabetes, 6.3% for patients with chronic respiratory diseases, 6.0% for patients with hypertension, and 5.6% for patients with cancer. In another study in which the estimation was adjusted for censoring, demography, and missing data, the fatality rate was 1.38% for COVID-19, 13.4% for patients ≥80 years old, 6.4% for patients ≥60 years old, and 0.32% for patients <60 years old. Patients aged 0–9 years old had the lowest fatality rate of 0.003% among all age groups.^26^

In China, most if not all of the children with SARS-CoV-2 infection had traceable history to either family members or recent contact with infected individuals.^27,28^ Children usually had mild symptoms. In a series of 2143 cases of Chinese pediatric patients (median age: 7 years), >90% of children were asymptomatic or with mild-to-moderate disease.^29^ Compared with adult patients, infected kids were less likely to have fever (children: 36%; adults: 86%), cough (children: 19%; adults 62%), and severe disease, including pneumonia (children: 53%; adults: 95%), elevated C-reactive protein (children: 3%; adults: 49%), and other severe disease types (children: 0%; adults: 23%).^13^

The major cause of death in COVID-19 patients is respiratory failure.^30,31^ In a retrospective study of 113 deceased patients, older people, male, patients with hypertension or other cardiovascular comorbidities (and with signs of myocardial damage), patients with hypoxemia-related symptoms, and patients with multiple organ dysfunction were at a higher risk to develop respiratory failure and die than others.^14^

Clinical parameters during hospitalization, which might be associated with high fatality, include markers of significant inflammation like leukocytosis, lymphopenia, elevated C-reactive protein levels, and elevated lactic dehydrogenase levels, as well as the appearance of clinical complications.^32^ In a retrospective study of 52 severely ill patients, 61.5% died within 28 days since diagnosis, and the median survival time of patients, from entering intensive care unit (ICU) to death, was 7 days. Nonsurvivors are mostly older patients (>65 years old) who usually have comorbidities.^33^ In another retrospective analysis of 78 patients, factors related to disease progression and poor prognosis included advancing age, history of smoking, high maximum body temperature at admission, evidence of respiratory failure, significant reductions in serum albumin levels, and elevated C-reactive protein levels.^34^ Thrombocytopenia was reported to be associated with severe illness and death, and also related to the development of disseminated intravascular coagulation (DIC).^35^ Other factors related to poor prognosis include a high Sequential Organ Failure Assessment scores and d-dimer levels >1 μg/L (a marker of DIC), another marker of coagulation system involvement. Importantly, these severely ill patients continued to be infectious, shedding virus till they died, highlighting the infection risk to the medical professionals.^2^

To predict the outcome of COVID-19 is of vital clinical importance to better allocate medical resources and provide individualized treatment for patients. The availability of clinical characteristics and parameters with potential prognostic implications will be of value in this respect and a number of institutions are conducting research in this direction.

### Latest data from other countries

In the United States, the epidemiology data are evolving rapidly. By the end of 2020, there were 19.66 million confirmed SAR-CoV-2 infection with >340,000 deaths.^36^ The State of New York conducted a serology test for antibodies to SARS-CoV-2 and found that 14–20% of New York City citizens (transit workers: 14.2%; citywide test: 19.9%) were positive, compared to ~3% in other parts of the State, with an overall ~12% of the tested subjects being seropositive for antibodies to SARS-CoV-2 (information released from New York Governor and New York City Mayor Office). Due to the fact that the results of the serology tests cannot be viewed alone without the other clinical and molecular assay data, the prevalence of COVID-19 in the New York State might even be underestimated. In the USA, the relationship between the fatality rate of the COVID-19 disease and age is well established. COVID-19 patients ≥85 years old have the highest fatality rate (10–27%), followed by 65–84 years old (3–11%), 55–64 years old (1–3%), and 20–54 years old (<1%), and ≤19 years old (<0.1%). Patients aged ≥65 years old account for 80% of deaths in the USA. Among the patients admitted to the ICU, 7% were patients ≥85 years old, 46% were 65–84 years old, 36% were 45–64 years old, and 12% were 20–44 years old, again confirming that seniors were the most vulnerable.^37^ As was reported in a hospital in Washington State, the mortality rate of critically ill patients in the ICU was 67% and most of them had underlying diseases, typically congestive heart failure and chronic kidney disease. In the same geographic region, the fatality rate of COVID-19-infected residents in a long-term care facility in Washington was 34%.^38,39^

Europe is also impacted substantially. As of today, Spain, Italy, UK, France, Germany, and Turkey all had >2.5 million confirmed infections (and they are all in the top 10 countries with the highest number of infected subjects) and with a mortality rate of 310 to 1606 per million. Russia and Iran also had a large number of infections, with 3.9 and 1.4 million confirmed infections and a mortality rate of 512 and 688 per million population, respectively.

In Latin America, Brazil at present has 9 million confirmed infections and a mortality rate of 1066 per million.

The overall fatality rate of COVID-19 appears to be lower (about 2%) than that of SARS (estimated to be 10%) and Middle East Respiratory Syndrome (MERS) (estimated to be 37%).^1^ Despite the lower fatality rate, the high infection rate had driven COVID-19 to more fatalities than the total of SARS and MERS combined.^40^

## Outbreak prediction and preventive measures

As mentioned above, several research teams have modeled the epidemiological data to forecast the potential spread of SARS-CoV-2 infections either locally or globally.^15,16,41^ Effective communications and collaborations among various countries have allowed testing and validating various hypotheses. Importantly, some control measures were implemented by various governments and their impact was assessed via population infection kinetics.

We have established a four-compartment model to determine the impact of various control and mitigation measures, including quarantine, lockdown, social distancing, and the general use of facemask, on the kinetics of infection in a population by comparing data either before or after the implementation of these measures and also between countries when different measures were implemented.^17,42–44^ This model considered both social interaction factors and viral transmissibility factors.^16^ The social interaction factors include per capita contact rate and also the infection rate upon contact, which can be modified by public policies, including reducing the social contact, or per capita contact rate by quarantine, lockdown, and social distancing, and also reducing the infection rate upon contact per general use of facemask. When this model was first established, we assumed similar viral transmissibility across virus variants recognizing that any RNA virus is known to have significant genetic heterogenicity for their survival generated through the lack of proof-reading activities of the viral RNA-dependent RNA polymerase. Recently, some of the variants have been shown to have increased transmissibility and, therefore, further revision of the model is warranted. Based on the analyses of this earlier mathematical model, the impact of social interaction factors (in particular, the per capita contact rate and the infection rate per contact) can be affected by public policies and recommendations (e.g., lockdown, the general use of facemask) imposed on a population or global scale. As mentioned earlier, some of the most recently identified SARS-CoV-2 variants isolated from South Africa were shown to have a higher infectivity clinically. Molecular characterization showed that amino acid substitutions on the spike (binding to the host cell receptor) and other viral proteins may be responsible for this viral evolution, which will always be in the evolutionary direction to become more infective and replication efficient. The mathematical model will certainly need to be adjusted when the viral transmissibility and viral replication efficiency (which may potentially reflect viral load) data are available.

This new knowledge, together with our understanding of infection control, will certainly impact the decision process of the lockdown exit, the importance of continuous monitoring, and also the reimplementation of some of these policies when there is a second wave of infection. Our knowledge in public health, in particular, infection control and mitigation measures, and the infection modeling knowledge, has advanced substantially recently, and hopefully, this will better prepare us for any future epidemics/pandemics.

## Virology and pathogenesis

The viral genomic organization has been published and we expect that more virology information will be available soon. It is important to note that SARS-CoV-2, like other RNA viruses, has substantial genetic variability due to the lack of proof-reading activities of viral RNA-dependent RNA polymerase, which from an evolutionary standpoint is critical for viral adaptation and its “survival.” A study by Forster showed three different genotypes of SARS-CoV-2 and with the type being considered to be ancestral type using a closely related bat coronavirus (with 96.2% homology) as the root.^45^ The A and C types are found mainly outside China, whereas type B is the most common type in East Asia. Although the sampling bias, the assumption/method of rooting, and the use of median-joining network may represent challenges in their analyses and conclusions, the genetic variability and the important role of phylogenetic network/tree analyses in our further understanding of this virus is certain. More studies in the foreseeable future will provide better insight into the classification and evolution of this virus.

The genetic variability of RNA virus will pose clinical challenges. First, the variability of the amino acid sequences in the spike protein is for host cell receptor binding, and whether any of the genotypic variations can lead to “escape” of the humoral neutralizing effect in recovered patients or vaccinees will have a significant clinical impact (see the South African variant below). Second, the virus can also evolve to be more replication efficient. Third, some of the viral proteins may evolve into mediators that may prevent the host immune system from recognizing or attacking infected cells, thus allowing infected cells to escape from the host immune attack/elimination and to generate more viruses. Finally, when antiviral therapy targeting a viral protein is available, the virus may mutate and “escape” and develop drug resistance.

Studies have confirmed that the SARS-CoV-2 binds to the human host receptor, the hACE2, suggesting that it has a similar tissue tropism as the SARS virus.^46,47^ As hACE2 is mostly expressed in type II alveolar (AT2) cells in lungs, endothelial cells in blood vessels, gastrointestinal epithelial cells, and hepatocytes, therefore, this explains the frequent incidence of pneumonia, the observation of vasculitic features, and also the detection of viral RNA and antigens in the feces. Previous studies based on single-cell RNA-sequencing data showed that hACE2 is expressed in the lung, heart, esophagus, gastrointestinal tract, liver, kidney, and bladder, consistent with the damages to these target organs observed in the SARS-CoV-2-infected patients.^48^ The high-level hACE2 expression in type II alveolar cells may explain the rapid onset and severity of pneumonia in some COVID-19 patients. Smoking has been shown to increase the lung gene expression of hACE2.^49^

Importantly, compared with SARS-CoV, the unique structural features of the SARS-CoV-2 spike protein receptor-binding domain (RBD, which enables SARS-CoV-2 to bind to the host cell receptor) provided a higher binding affinity towards hACE2. Furin protease cleavage sites were also found in the spike protein of SARS-CoV-2, which was not present in other SARS-like coronaviruses.^50^ In other coronavirus infections, the spike protein was found to downregulate hACE2, leading to excessive accumulation of angiotensin-II toxicity, which in turn may contribute to the progression to acute respiratory distress syndrome and fulminant myocarditis.^51^

Given the high level of genetic variability and virus adaptability, it will not be surprising that new SARS-CoV-2 variants will emerge upon selective pressure from either antiviral treatment of the host immune selective pressure triggered by either the infection or the newly approved vaccines. Since September 2020, there were three viral variants that have raised concerns. On 21 January 2021, the UK New and Emerging Respiratory Virus Threats Advisory Group (NERVTAG) issued a paper outlining the results from several preliminary analyses of B.1.1.7.^52^ The variant, which is highly transmissible, was initially identified in the south of England in September 2020. It has since spread to dozens of countries around the world. Molecular characterization showed that it has 17 mutations with amino acid substitutions/deletions in its genome, including eight in the spike protein (including the deletion of amino acids 69–70, which is believed to lead to conformation changes of the spike protein), which forms the basis of a number of the COVID-19 vaccines. NERVTAG concluded that there was a “realistic possibility” that infection with B.1.1.7 is associated with an increased mortality, compared with infection with the parental virus. The group stressed that its assessment was based on limited preliminary data, and even if confirmed, the overall risk of death would still be low. Another extremely infectious variant, P.1, has been circulating in Brazil since mid-2020 and was believed to contribute to the surge of infections in the Brazilian Amazon. Recently, B.1.351 was identified in South Africa in late 2020 (with some samples dating back to October 2020) with a number of mutations similar to the UK variant, but does not contain the deletion of the amino acid 69/70), but with an amino acid substitution E484K, which may affect neutralization by some polyclonal and monoclonal antibodies.^53,54^ So far, there is no evidence that this variant has any impact on disease activity. This virus has spread to many countries including the USA (as of today, six cases identified in three different States), UK, China, and others. With this profile, there is a concern that the infected subjects who recovered may be susceptible to another round of infection and also the currently developed vaccines may not be able to protect against this variant, which will have a major impact on the evolution of this pandemic. The latest on the impact of this variant on vaccine efficacy will be discussed later in the vaccine section.

In February 2020, the first completed autopsy report of a deceased COVID-19 patient was released. The results showed an extensive inflammatory reaction with deep airway and alveolar damage, which are very similar to the pathological features of SARS and MERS. Electron microscopy examination of autopsy specimens showed the existence of large numbers of viral particles in alveolar epithelial cells. Gross pathology showed varying degrees of atrophy on all the lung lobes, and the cut surface showed decreased lung air volume with various degrees of consolidation. No obvious secretion retention was found in the trachea and the main branches of the bronchus. In the same report, pathological findings of limited lung autopsy of other COVID-19 patients were also provided. Most patients’ lungs, especially the middle and lower lung lobes, were adhered to the chest wall, suggesting inflammation of the peripheral lung tissue leading to the formation of adhesions. Microscopically, the main pathological changes in the lung were the increased number of macrophages in the tissue, serous fibrinous exudation (which could show up as ground-glass appearance in chest CT scans), accompanied by hemorrhage in some of the alveolar cavities, diffuse alveolar lesion, alveolar degeneration, and pulmonary consolidation. In some patients, a small number of alveolar cavities showed hyaline formation, type II alveolar epithelium hyperplasia with a widening of the alveolar space to various degrees, and interstitial fibrosis with lymphocytic infiltrates. In the small airways (mainly bronchiole and terminal bronchiole), there was mucus retention, and some had mucus plugs. Importantly, many patients had secondary bacterial infections, as evidenced by neutrophil-dominant inflammatory cell infiltrates in some of the lesions. A few patients had secondary fungal infections, as evidenced by the existence of fungal hyphae and spores in the lesions. These descriptions are consistent with the pathological changes of other viral pneumonia, and this can be compounded by secondary bacterial and fungal infections.^22,55^ These findings are critical for clinicians as they may need to consider covering the patients early with anti-infective and anti-fungal treatments once there are clinical suspicions of secondary infection in these patients.

**Fig. 1:**
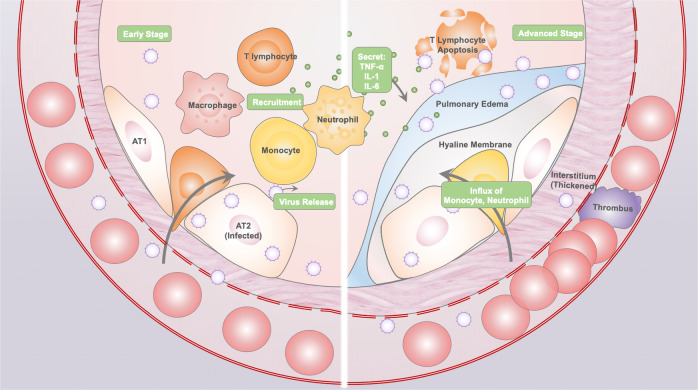
Immunopathogenesis of coronavirus disease 2019 (COVID-19) in early and advanced stage

In the early stage of infection, SARS-CoV-2 virus entering the targets cells, such as bronchial epithelial cells and AT2 cells, could induce a series of host immune response. Furthermore, inflammatory signaling molecules are released by infected cells and alveolar macrophages in addition to recruited monocytes, neutrophils, and T lymphocytes. In the advancing stage of infection, SARS-CoV-2 virus infects pulmonary capillary endothelial cells, triggering an influx of monocytes and neutrophils, killing T lymphocyte cells, and accentuating the inflammatory response.^56^ As a consequence, thickened interstitium, hyaline membrane formation, pulmonary edema, and activation of coagulation contributing to microthrombus formation even pulmonary thrombus may develop and appear (Fig. 1). The development of viral sepsis, referred to as life-threatening organ dysfunction, may further lead to multiorgan failure.

## Diagnostics for COVID-19

The detection of the viral nucleic acid sequence by either real-time reverse-transcription polymerase chain reaction (RT-PCR) analysis using viral-specific primers, nucleic acid next-generation sequencing (NGS), or other molecular tools is currently the gold standard for diagnosing SARS-CoV-2 infection. The current targets of SARS-CoV-2 sequence detection include three conserved gene sequences in the viral genome including the open reading frame (ORF), nucleocapsid protein (N) gene, and envelope protein (E) gene.^57^ The specimens for testing can be nasopharyngeal swabs, sputum, other lower respiratory tract secretions, blood, and feces. Yang et al.^58^ studied the different types of specimens from the respiratory tract (nasal swabs, throat swabs, sputum, bronchoalveolar fluid) in COVID-19 patients to evaluate the diagnostic accuracy based on a molecular diagnostic assay approved by the Food and Drug Administration (FDA) of China and found that sputum had the highest accuracy followed by nasal swabs. Another study tested the feasibility of detecting SARS-CoV-2 nucleic acid sequence in saliva, and SARS-CoV-2 sequence was detected in 91.7% (11/12) of COVID-19 patients’ saliva.^59^ Despite the high sensitivity of the RT-PCR assay, the type of sample collected, sample storage method, the time needed for transportation to viral RNA extraction, and the reagents used for extraction can all contribute to the variability of the detection sensitivity. On the other hand, assays that were contaminated by the viral sequence DNA amplimers generated from other samples could create false positivity given the molecular amplification involved in the diagnostic system. Therefore, a reliable one-step device at low cost and reasonable efficiency is urgently needed. Certainly, a rapid point-of-care assay that can diagnose COVID-19 and other viral and bacterial pneumonia at the same time will be a very useful tool going forward. It will be reasonable to assume that SARS-CoV-2 is here to stay and can concurrently infect an individual with other respiratory pathogens including influenza, parainfluenza, rhinovirus, and other viral and bacterial pathogens.

Serologic assays for the detection of viral antigens, as well as IgG and IgM antibodies against the viral antigens, have also been developed for SARS-CoV-2 infection. Obviously, the detection of SARS-COV-2 viral antigen will be more rapid and can be a point-of-care test, but the sensitivity will be lower than that of the molecular PCR-based assays.

In a study that evaluated the respiratory viral load and serum antibody response in patients with COVID-19, saliva virus load was the highest in the first week after the onset of symptoms, which then declined over time. Most patients develop antibody responses (both IgM and IgG) on/after 10 days of symptoms.^60^ Serum antibody levels did not correlate with clinical activity. In a more recent study, we showed that IgG and IgM appeared at around the same time and with a specific serology assay. We also showed that there was a high proportion of people in China who had exposure to SARS-CoV-2-developed antibodies to the virus (up to 3.4%) but remained asymptomatic.^61^ As this is a novel virus, assuming that the capture antigen only contains epitopes specific to this virus, the seroprevalence will be reflective of the cumulative attack rate of this virus in the first season. There are a large number of such assays manufactured by different companies, but concerns remain on the performance (i.e., specificity, sensitivity, accuracy, reproducibility) of these assays, on top of the timing of the collection of the samples in the course of infection for a particular subject.^22,62–64^

One key question going forward is how long the antibody response will last in infected subjects or vaccinees. If the humoral response does not last long, there is a need for booster doses and this question can only be addressed when infected subjects and vaccinees are followed up both by serology and clinically.

## Application of artificial intelligence (AI) and internet technologies

### AI-aided radiologic diagnosis/screening system for COVID-19

Computed tomography (CT) of the chest is now considered as the primary imaging method of COVID-19 pneumonia due to high accuracy. In a meta-analysis of 50,466 inpatients, up to 97% of COVID-19 patients had abnormal chest CT.^65^ Some reports indicated that CT scan could identify COVID-19 earlier even when compared to SARS-CoV-2 viral RT-PCR test in some patients.^66^ Also, chest CT imaging abnormalities can occur in patients with mild or no symptoms.^67^ In CT scan, most patients showed multiple ground-glass shadows and infiltrating shadows on bilateral lobules and subsections, while in severe cases, pulmonary consolidation may occur. Atypical features seem to be more common late in the disease.^63^ Since the outbreak of the virus, as the burden placed on radiologists to read radiographic images skyrocketed, AI was considered as a tool to assist radiologists in COVID-19 pneumonia diagnosis.

**Fig. 2:**
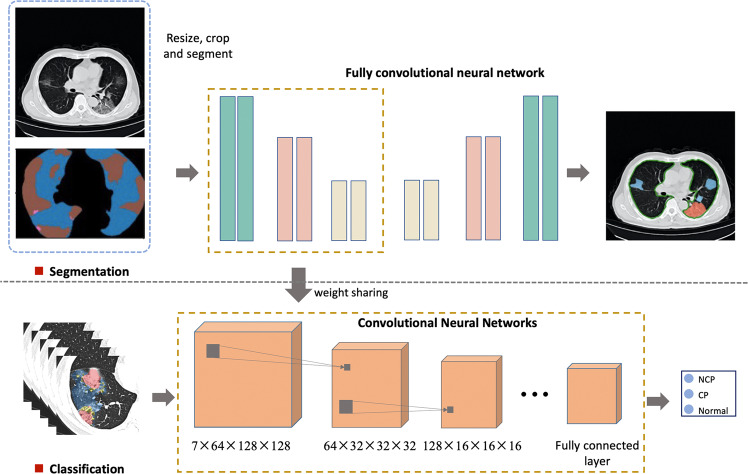
Illustration of network architectures of the proposed AI diagnostic system for COVID-19

We and other colleagues have established AI systems for accurate diagnosis of COVID-19 pneumonia based on CT parameters through deep learning.^68,69^ Using a large CT database from 3777 patients, we developed an AI system that can diagnose novel coronavirus pneumonia (NCP) and differentiate it from other common pneumonia (CP) and normal controls (Fig. 2). With the combination of image-based and quantifiable clinical parameters, they also analyzed the relationship between imaging features and clinical markers and provided an AI model in prognostic prediction on progression to critical illness.

Obviously, with the sophisticated implementation procedures and high-cost chest CT scans are not suitable as a frontline tool. To develop a comprehensive system to help combate COVID-19, we are also trying to develop an AI system for chest X-ray as a fast, frontline screening tool for the diagnosis of viral pneumonia, conceivably even before viral molecular tests results are available. Preliminary results have been encouraging. This can be of utmost importance to public health in a pandemic situation as this is a first-line assessment available in most healthcare centers, requiring radiographers only, with a quick turn-around time.

In general, AI can be very useful in the screening and management of COVID-19,^70^ including increasing the screening accuracy of suspected cases, predicting the survival of critically ill patients, providing an optimal treatment plan, and in the screening of antiviral drugs. AI-powered robotic systems can also perform tasks for disinfection and support social distancing that is usually performed by humans, and thus reducing exposure risks.

### Application of mobile internet in healthcare

With the rapid development of wireless internet technology and the increasing number of mobile phone users, Mobile Health technology has emerged as a potential solution to healthcare delivery for people with chronic diseases.

An AI-based medical assistant system can provide online analytic assistance to hospitals and clinics in analyzing the patient’s history, symptoms, and signs, imaging, laboratory blood tests, and even correlate with the latest epidemiology data (e.g., latest prevalence of COVID-19 in the area and areas that the subjects have traveled to in the last month) in risk assessment and identify the suspected COVID-19 patients, thus providing decision-making reference for the healthcare providers. During the COVID-19 endemic in China, more than 190 public medical institutions and nearly 100 internet hospitals across China provided online free consultation services, this avoiding patient contact and direct hospital visits, and at the same time providing some routine patient care needs while avoiding the risk of nosocomial COVID-19 infection.

### AI-assisted public health initiatives

Big data from phone records, travel records, and social media data can provide travel patterns and trajectories of patients with suspected infection, which can be used to track patient’s close contacts, and forecasting outbreaks.

The Allen Institute of AI, in collaboration with leading research institutions, has released an open research database with weekly updates on the COVID-19 to accelerate new research projects that need real-time data.^71^ Information application platforms such as geographic information systems, mapping dashboard, and case-tracking applications can enable online real-time or near real-time monitoring of patient trajectories and social media response to disease spread. Based on population travel data prediction risk maps and the trajectories of super spreaders and close contacts, the platforms can reveal the temporal and spatial distribution of COVID-19, and have been shown to be a timely and effective monitoring tool to track outbreaks and recommend appropriate government responses.^72^ The visual and interactive global epidemic map developed by the research team at Johns Hopkins University in the USA is currently the most widely used COVID-19 epidemic surveillance.^73^

For the epidemiology data, COVID-19 data in China, USA, Canada, and Australia, are collected at the provincial/state level, while for other countries, data are collected at the country level. In China, the National Health Commission, in collaboration with industry, developed an “intimate contact measurement” application platform in February 2020, and through this tool, the public can check if they were/are in close contacts with known infected COVID-19 patients by entering their name and ID number.^70^ Obviously, privacy issues may not allow this to be implemented in other countries.

## Treatment of COVID-19

In response to this serious global pandemic, the world’s biomedical/pharmaceutical establishments were unleashing an unprecedented response to the finding of safe and effective treatment strategies for COVID-19. Within 1 year, an antiviral drug, Remdesivir, and monoclonal antibodies against SARS-CoV-2, including bamlanivimab, and also the combination use of casirivimab and imdevimab were approved by the United States FDA and other regulatory authorities under Emergency Use Authorization (EUA) for the treatment of COVID-19.

From a pathogenetic mechanism perspective, as the viral infection spread to the lungs quickly and the COVID-19 pneumonia lung damage appeared to be a consequence of the host’s immune response attacking the infected lung cells, some investigators considered the drastic immune reaction to the lung tissue having some resemblance to the cytokine storm syndrome observed either in the transplant setting or as an adverse event of the chimeric antigen receptor T cell (CAR-T) therapy. Therefore, current attempts in the design of new therapies are focused on either the viral infection with either known antivirals or known drugs found to have antiviral activity in cell culture experiments or cytokines with known antiviral effect including interferons, or the control of the immunopathogenesis using immunomodulators to allow the lungs a chance to recover. There are also investigators evaluating the role of Chinese herbal medicine, the use of passive immunotherapy using serum from convalescent patients with COVID-19 and the use of stem cells for lung cell regeneration.

Early efforts were focused on identifying existing drugs that might also show antiviral effect against SARS-CoV-2 replication. There were reports of a large number of known drugs that showed some antiviral activity against SARS-CoV-2, including chloroquine, ribavirin, interferons, lopinavir/ritonavir, and others.

As the first country to be affected by COVID-19 in a major way, most of the Chinese COVID-19 patients were on some type of clinical study protocol and such an approach has addressed clinically some of the early suggestions based on laboratory virus cell culture experiments.^1,74,75^

Chloroquine, an old anti-malarial drug with high lipid solubility, is known for its pH-dependent antiviral effect including coronavirus. Hydroxychloroquine in combination with azithromycin was also getting attention initially based on a small single-arm pilot study in France showing a reduction of viral load during therapy.^76^ Another study conducted in China (*n* = 62, patients with mild/moderate pneumonia) comparing hydroxychloroquine alone vs standard care at the time of the study also suggested a beneficial effect.^75^ In contrast, there were also a number of reports from Spain, UK, and USA, showing the lack of clinical benefit of hydroxychloroquine and azithromycin in COVID-19 patients.^77–79^ Also, this class of drugs is also known to induce QT prolongation and ventricular arrhythmia. Although there is no solid evidence that azithromycin treatment induces QT prolongation, there were reports of an excess of cardiovascular deaths associated with its usage (47 cardiovascular events per million completed courses of treatment) highlighting the potential risk involved. In fact, the American College of Cardiologists issued their opinion on 29 March 2020, suggesting that physicians should be aware of this potential complication. Given the clinical challenges, FDA issued an EUA on 28 March 2020 for the use of chloroquine and hydroxychloroquine in adults and adolescents for those for whom a clinical trial is not available or participation not feasible and required the mandatory reporting of adverse events to FDA Med-Watch. While controlled clinical studies were being conducted to evaluate the risk–benefit ratio of this treatment approach, FDA revoked the EUA on 15 June 2020, after they analysed the emerging scientific data, and determined that the legal criteria for issuing an EUA was no longer met.^80^

Ribavirin, in combination with interferon, was also used in some of the early clinical studies. Ribavirin is a viral-static agent in the cell-based assay. It is both an RNA-dependent RNA polymerase inhibitor and a viral mutagen (the sugar moiety of ribavirin is a pentose with hydroxyl groups in both 2′ and 3′ position and will allow integration into the RNA), which may cause replication catastrophe for the virus despite replication. Therefore, its effect as monotherapy may only be revealed in terms of clinical outcome instead of reduction in viremia, as reflected in the chronic hepatitis C studies. In addition, ribavirin showed strong synergistic activities with interferon and was used in combination to treat hepatitis C, another single-stranded RNA virus, in the late 1990s. The combination of ribavirin and recombinant interferon has also been shown to have potent efficacy in inhibiting MERS-CoV replication, another coronavirus.^81^ In the Chinese National Health Commission new coronavirus infection pneumonia diagnosis and treatment plan (trial version 7), one of the recommendations was to consider the use of intravenous ribavirin in combination with inhaled interferon-beta-1b as a possible treatment option for further evaluation.^82^ A prospective, controlled, multicenter clinical study (ChiCTR2000030922) of long-acting interferon plus ribavirin to treat COVID-19 was currently conducted in China to evaluate its clinical efficacy and safety.

Lopinavir–ritonavir was initially identified to have micromolecular IC-50 activity against SARS-CoV-2 in in vitro testing, offering hopes for clinical activities for this anti-HIV protease drug combination based on drug repurposing. A recent publication based on a randomized controlled study in 199 COVID-19 patients showed that the combination of lopinavir–ritonavir offered no clinical benefit in adult patients with severe COVID-19.^83^ In another published phase II study, triple combination treatment consisting of interferon-beta-1b, ribavirin, and lopinovir–ritonovir was compared with lopinovir–ritonovir alone (control arm) and showed safety and superior efficacy in alleviating symptoms and shortening the duration of viral shielding and hospital stay in patients with mild-to-moderate COVID-19 (ClinicalTRials.gov: NCT04276688).

Remdesivir is a potent RNA-dependent RNA polymerase inhibitor initially developed for the Ebola and Marburg viruses, which was found to have a good effect against respiratory syncytial virus, Junin virus, Lassa Fever virus, and coronaviruses, including SARS and MERS, and has recently also been shown to have good inhibitory activity against SARS-CoV-2. It is a prodrug of an adenosine analog given intravenously. Early clinical experience in the US showed that remdesivir is effective in reducing the SARS-CoV-2 viral load without significant adverse events. In China, two clinical trials are currently conducted to evaluate the efficacy of remdesivir in COVID-19 patients with the mild-to-moderate disease (NCT04252664) and severe critically ill patients (NCT04257656). In the initial series of 12 US patients with COVID-19 in the USA, the CDC reported that three patients received remdesivir as part of the expanded access program and noted gastrointestinal side effects and elevated liver enzymes in these patients, highlighting the importance of addressing both clinical efficacy and potential adverse events in the currently ongoing phase III studies.^84^ Another phase III study on the use of remdesivir in hospitalized patients with COVID-19 who received remdesivir had a 31% faster time to recovery than those who received placebo (*p* < 0.001). Specifically, the median time to recovery was 11 days for patients treated with remdesivir compared with 15 days for those who received placebo. Results also suggested a survival benefit, with a mortality rate of 8.0% for the group receiving remdesivir vs 11.6% for the placebo group (*p* = 0.059). This drug received FDA EUA for the treatment of suspected or laboratory-confirmed COVID-19 in adults and children hospitalized with severe diseases 2 days after the data were available. Based on the same dataset, remdesivir was also approved in Japan via an exceptional approval pathway 8 days later.

Another board-spectrum antiviral, umifenovir, which inhibits membrane fusion of the virus to the host cells and registered for use on the influenza indications in Russia, was also found to have an effect against SARS-CoV-2 in virus cell culture assay. A multicenter randomized controlled trial (ChiCTR2000029573) is underway to evaluate the efficacy of umifenovir in combination with lopinavir/ritonavir vs the three drugs together with interferon.

Passive immunotherapy using plasma from COVID-19 convalescent patients were also evaluated early on as a treatment option. A small pilot study (5 patients) in China showed that after receiving passive immunotherapy plasma, the patient’s viral load decreased rapidly, and the patient’s clinical symptoms improved, highlighting the viral neutralizing activities in the convalescent patients’ plasma.^85^ On 27 March 2020, Houston Methodist Hospital announced that they have obtained an emergency investigational new drug application to evaluate this convalescent plasma therapy in critically ill COVID-19 patients in the USA. Although this approach may have some scientific merits, the potential adverse reactions of plasma therapy should not be overlooked. In addition, the standardization process in terms of the dose, treatment regimen, and its long-term impact on the recipient’s immune response development have not been established, which should be addressed in randomized clinical studies.^86^

Along the same line, a number of pharmaceutical and biotech companies developed monoclonal antibodies against the spike protein of SARS-CoV-2, which contains the RBD that binds to the host cell receptor hACE2. The first antibody tested was bamlanivimab (LY-CoV555). In a phase 2 study, studying three doses of the drug vs placebo (465 patients in four arms) in patients with mild-to-moderate COVID-19 and within 10 days of symptom onset, there was a reduction in the nasopharyngeal SARS-CoV-2 level with the high-dose group, and a reduction in pre-specified endpoint of COVID-19-related hospitalization, emergency department visit, or death (6.3% in placebo vs 1.6% overall for the three doses group). On 9 November 2020, FDA issued an EUA based on the phase 2 data. However, another study of the same antibody did not meet the primary endpoint for COVID-19 patients who were hospitalized. FDA did emphasize in their press release that this antibody was not authorized for hospitalized COVID-19 patients and that the use of this antibody might be associated with worse clinical outcomes in the hospitalized patients with COVID-19 requiring high-flow oxygen or mechanical ventilation. In a phase III study, another monoclonal antibody against SARS-CoV-2 spike protein epitope, etesevimab (LY-CoV016), was evaluated in combination with bamlanivimab, and on 26 January 2021, there was an announcement that in the 1035 patients with mild and moderate COVID-19 and within 10 days of symptom onset (non-hospitalized), this combination use of monoclonal antibodies resulted in a 70% reduction (7.0% in placebo vs 2.1% in treated group) in the pre-defined events (defined as COVID-19-related hospitalizations and deaths).

On 21 November 2020, 12 days after the EUA of bamlanivimab was issued, FDA also issued an EUA for casirivimab and imdevimab (REGN10933 and REGN10987), two monoclonal antibodies recognizing two different epitopes on the SARS-CoV-2 spike protein RBD. This authorization was based on a phase 2 study involving 799 non-hospitalized adult patients with mild-to-moderate COVID-19 symptoms, and similar endpoint results as bamlanivimab were achieved. On 29 December 2020, encouraging initial results (that passed the futility analysis) were also announced for the use of this antibody cocktail in hospitalized patients on low-flow oxygen. The immune status of patients on entering the trial was a strong predictor of viral load and outcome. Patients seronegative for antibody against SARS-CoV-2 had significant viral load drop and passed the futility test for lower risk of death or requiring mechanical ventilation (~50% reduction), based on an interim post hoc analysis. This study is still ongoing.

Another approach is to prevent severe lung disease development related to the host immune attack. Alveolar lavage fluid from COVID-19 patients based on single-cell sequencing technology showed that macrophages may play an important role in the pathogenesis of COVID-19.^87^ Macrophages are important effector cells for interleukin-6 (IL-6). The elevated IL-6 also showed a similar profile as observed in bone marrow transplant patients and patients with cytokine storm syndrome after CAR-T therapy. Tocilizumab is a humanized IgG_1_ monoclonal antibody that can specifically bind to soluble or membrane-bound IL-6 receptors and blocks the signaling pathways of both IL-6 and granulocyte–macrophage CSF (GM-CSF), and this reduces the systemic inflammatory response.^88^ A multicenter randomized controlled clinical study is already underway to evaluate the efficacy and safety of tocilizumab in the treatment of patients with moderate to severe and critical illness in China (registration number: ChiCTR2000029765). There are studies ongoing in other countries as well. On 3 February 2021, FDA issued a treatment guideline on tocilizumab and other IL-6 inhibitors. The panel summarized that initial studies evaluating the use of IL-6 inhibitors for the treatment of COVID-19 produced conflicting results. Many trials were limited by low statistical power, heterogeneous study populations with varying degree of disease severity, and/or low frequency of concomitant use of corticosteroids, which has become the standard of care in patients with severe or critical COVID-19. Based on available information, the FDA panel determined that (a) for patients who are within 24 h of admission to the ICU or who require invasive or noninvasive mechanical ventilation or high-flow oxygen (>0.4 FiO_2_/30 L/min of oxygen flow), there are insufficient data to recommend either for or against the use of tocilizumab (or sarilumab, which is also an anti-IL-6) for the treatment of COVID-19, and (b) for patients who do not require ICU-level care or who are admitted to the ICU but do not meet the critical need listed in (a), tocilizumab or sarilumab should not be used apart from a clinical trial setting.^89^

There are also other investigators who are evaluating other immunomodulatory approaches, including antibody against CCR5, antibody against GM-CSF, antibody against vascular endothelial growth factor, and immunostimulants anti-PD1 and thymosin. No date from any large clinical studies on these modalities is currently available.

Along the same line, it will be tempting for investigators to evaluate the effect of glucocorticoids in COVID-19 patients to reduce the excessive host immune attack against the SARS-COV-2-infected cells. However, one should also note that steroids may impact the immune system and reduce the host immune response against the virus with the induced immunosuppression. In addition, steroids like dexamethasone tend to increase clotting factor and fibrinogen concentrations, and in a procoagulant state in severe COVID-19, there is the potential that steroid use may also induce damages.^90^ On 17 July 2020, the preliminary report of an open-label study on the use of dexamethasone in 6425 hospitalized patients (2104 on dexamethasone plus the standard care, 4321 on usual care) with COVID-19 showed that the incidence of death was lower among patients receiving invasive mechanical ventilation (29.3% in the dexamethasone group vs 41.4% usual care group) and also patients receiving oxygen without invasive mechanical ventilation (23.3 vs 26.2%) in the dexamethasone-treated group.^91^ The beneficial effect was not seen in patients who were not receiving respiratory support. As of today, the use of dexamethasone 6 mg for up to 10 days in hospitalized COVID-19 patients is recommended. In China, the current practice is that glucocorticoids should only be considered if the following four conditions are met: (1) adults (age ≥18 years); (2) SARS-CoV-2 infection confirmed by PCR or serum antibodies against the virus; (3) symptoms (including fever, cough or other related infection symptoms) occurred within 10 days, with radiologically confirmed pneumonia and with rapid progress; and (4) the patient’s blood oxygen saturation is ≤93% or significant shortness of breath (breath rate ≥30 breaths/min) or with PaO_2_ ≤ 300 mm Hg. In addition, the use of glucocorticoids should follow the principle of “low dose with a short course”^88^ and the aim of using the steroids to reduce the edematous component that impedes patient’s oxygenation. A randomized controlled study (ChiCTR2000029386) based on the above principles evaluating the efficacy and safety of glucocorticoid therapy in severe COVID-19 with low tissue oxygenation is ongoing in China.

In China, stem cell therapy was also evaluated in patients with severe/critical COVID-19. The principle is to provide fresh stem cells to assist the various organs, in particular the lung, to have a better chance to recover.^32^ At the time of writing of this report, there are 22 COVID-19 stem cell therapy clinical studies registered in the Chinese Clinical Trial Registry.

With regards to the general supportive care for these patients, there are four major areas for attention based on all the clinical reports and our personal experiences. The most important is the pulmonary function support. Pneumonia is the most common feature of severe COVID-19.^92^ For mild-to-moderately severe COVID-19 patients with hypoxemia, supplemental oxygen therapy, including high-flow nasal catheter oxygen therapy should be used when needed. For severe/critically ill patients with respiratory distress, noninvasive or invasive mechanical ventilation, or even extracorporeal membrane oxygenation should be considered.^93,94^ The second is the support of the renal function. Three clinical studies have indicated that the proportion of patients receiving continuous renal replacement therapies (CRRT, i.e., dialysis) is moderate-to-severe COVID-19 patients was 7–9%. In the ICU settings, CRRT was usually required in 5.6–23.0% of all patients.^95–97^ The third aspect is the coagulation profile. With rapid tissue damage induced by the virus, an abnormal coagulation profile suggestive of low-grade DIC is common and was observed in ~20% of all COVID-19 patients, and in nearly all severe/critically ill patients.^88^ A study is ongoing to evaluate the efficacy and safety of enoxaparin sodium in the treatment of coagulation disorders in hospitalized COVID-19 patients (ChiCTR2000030701). Finally, bacterial and fungal secondary infection is another important factor to consider. In a study of 99 patients with COVID-19, one patient had *Klebsiella pneumonia*, *Acinetobacter baumannii*, and *Aspergillus flavus* detected simultaneously and repeatedly in the sputum culture. One patient had *Candida glabrata* and three patients had *Candida albicans* secondary infection.^75^ Therefore, it is essential to watch out for secondary infection in the presence of COVID-19 pneumonia.

## SARS-CoV-2 vaccine development

A considerable number of SARS-CoV-2 preventive vaccine projects were initiated shortly after the reporting of this virus, including technologies that generate inactivated virus vaccine, viral protein subunits vaccine, messenger RNA (mRNA) vaccine, DNA plasmid vaccine, and recombinant human adenovirus type 5 (rAd5) or simian adenovirus type 26 (rAd26) expressing SARS-COV-2 spike protein, non-viral replicating vector expressing SARS-CoV-2 protein vaccine, and also replicating viral vector expressing SARS-CoV-2 protein vaccine. So far, there have been at least 30 announced vaccine projects globally, and vaccines derived from mRNA, expression using recombinant adenoviral vectors, and inactivated virus have already gained regulatory approvals in certain countries.^98–105^ The major COVID-19 candidate vaccine platforms were listed in Table 1.

**Table 1:** Major CoVID-19 candidate vaccine platforms in clinical evaluation

Vaccine name	Vaccine platform	Developer	Clinical trial phase	Clinical trial registrations
BNT162b1/BNT162b2	RNA-based vaccine	Pfizer-BioNTech, Fosun Pharma	Phases I–III in USA, Germany, and China	NCT04368728, NCT04380701, NCT04523571
mRNA-1273	RNA-based vaccine	Moderna, NIAID	Phases I–III in USA	NCT04470427, NCT04405076, NCT04283461
INO-4800	DNA plasmid vaccine	Inovio Pharmaceuticals, International Vaccine Institute	Phases I–III in USA	NCT04447781, NCT04336410
GX-19	DNA plasmid vaccine	Genexine Consortium	Phases I and II in South Korea	NCT04445389
ChAdOx1 nCov-19 (AZD1222)	Adenovirus vector, non-replicating	University of Oxford, AstraZeneca	Phases I–III in UK, South Africa, USA and Brazil	NCT04324606, ISRCTN89951424, EudraCT2020-001228-32, PACTR202006922165132, EudraCT2020-001072-15
Ad26.CoV2-S	Adenovirus vector, non-replicating	Johnson & Johnson	Phases I–III in USA and Belgium	NCT04436276 NCT04505722 NCT04535453 NCT04509947
Ad5-nCoV	Adenovirus vector, non-replicating	CanSino Biologics Inc., Beijing Institute of Biotechnology	Phases I and II; phase II studies in China and Canada	ChiCTR2000031781, ChiCTR2000030906, NCT04341389 NCT04313127
Gam-COVID-Vac	Adenovirus vector, non-replicating	Health Ministry of the Russian Federation	Phases I–III in Russia	NCT04530396 NCT04436471 NCT04437875
PiCoVacc	Inactivated SARS-CoV-2	Sinovac Biotech	Phases I–III; phase III in China and Brazil	NCT04456595, NCT04383574, NCT04352608
COVID-19 vaccine	Inactivated SARS-CoV-2	Sinopharm, Wuhan Institute of Biological Products Co. Ltd	Phases I–III in China	ChiCTR2000034780, ChiCTR2000031809
BBIBP-CorV	Inactivated SARS-CoV-2	Sinopharm, Beijing Institute of Biological Products Co. Ltd	Phases I–III in China and United Arab Emirates	ChiCTR2000034780, ChiCTR2000032459
SCB-2019	Protein subunit	Clover Pharmaceuticals,GlaxoSmithKline, Dynavax	Phase I in Australia	NCT04405908
NVX-CoV2373	Protein subunit	Novavax	Phases I–III in Australia, USA and UK	NCT04368988 NCT04583995 NCT04533399

To conduct experiments with SARS-CoV-2 requires a laboratory environment of at least Biosafety level 3. The use of mRNA in a vaccine is innovative and relatively safe. As it is synthetic, the path of product development will be much faster. Traditionally, a vaccine will take 10–15 years to be confirmed safe and commercially mature. But in such a pandemic context, the public and governments demand that an effective preventive vaccine be available as soon as possible. Regulatory authorities have also expressed their willingness to expedite the regulatory review process under the EUA path.

### mRNA vaccines

The first two vaccines that advanced quickly to clinical studies were based on the mRNA technology.

The first mRNA COVID-19 vaccine was received by FDA EUA on 10 December 2020. Based on an efficacy trial involving 44,000 volunteers, only eight people who got two shots of the vaccine spaced 21 days apart developed the disease, as compared with 162 participants in the placebo group, giving a COVID-19 disease efficacy of 95%. Severe disease occurred in nine placebo recipients, but in only one vaccine recipient there was a temporary need for oxygen and was not hospitalized. This vaccine, named Pfizer-BioNTech COVID-19 vaccine (BNT162b2), has been approved by health agencies for application in subjects 16 years or older.^106^ The challenge is that this vaccine needs to be stored at −80 °C during transportation, which is hard to achieve during the delivery and distribution.

The second mRNA COVID-19 vaccine (mRNA-1273) was approved by FDA EUA a week later on 18 December 2020.^107^ The efficacy trial was conducted based on ~30,000 volunteers, resulting in an efficacy of 94% with 11 volunteers in the vaccine group and 185 subjects in the placebo group developing COVID-19. Severe COVID-19 illness occurred in 30 recipients in the placebo group and only one in the vaccine group. This vaccine has been approved for those aged 18 years and older; the standard for the storage condition of this vaccine is −20 °C.

For both the aforementioned vaccines, FDA requested the developers to prepare a long-term plan of follow-up in pharmacovigilance on a public scale to complete the safety profile, while acknowledging the effectiveness of both vaccines in preventing symptomatic COVID-19 disease occurrence. There are no data available yet as to whether the mRNA vaccine also prevents infection. In a survey in Israel, the Israeli Ministry of Health^108^ reported that of the 750,000 fully vaccinated people over 60 years old, only 531 (0.07%) were tested positive for COVID-19 so far, and 38 were hospitalized, with symptoms ranging from moderate to critical. Most experts believe that a proportion of the vaccinees may still get COVID-19 and be likely to spread the virus. Therefore, we should still recommend the vaccinees to use a facemask and observe social distancing. Despite these limitations/cautions, the benefits of the mRNA vaccines are significant and outweigh the concerns, and they have been approved in a number of countries already.^109,110^

With the recent identification of a viral variant in South Africa, in which its mutation in spike protein (E484K) was found to affect neutralization by some polyclonal and monoclonal antibodies, there was a concern as to whether these early mRNA vaccines will still be effective. Both companies have tested this question in laboratory studies. The sera from vaccines for both the mRNA vaccines were found to still be effective in neutralizing the variant, but with less activity compared to the wild-type SARS-CoV-2 virus. The studies have not been peer-reviewed. As these are synthetic mRNA vaccines and can be adjusted for the variant sequence quickly, they are planning to develop a new vaccine targeting the South African variant for further testing.

### Recombinant adenoviral vectors expressing SARS-CoV-2 spike protein/RBD

A number of other institutions/companies in China, UK, Russia, and USA developed recombinant adenoviral vector expressing the COVID-19 genes as vaccines. China has a version based on the human adenovirus vector (rAd5 backbone). United Kingdom (Oxford University) has a version (code name AZD1222) that is based on a simian adenoviral vector. A multinational company also has a similar vaccine based on Ad26 backbone (code name Ad.26.CoV2.S or JNJ-78436725), which have data suggesting that one dose may be enough. Russia has a COVID-19 vaccine (code name Sputnik V) that uses two recombinant adenovirus backbone, with one dose using the rAd5 and the other dose using the rAd26 to reduce the interference by the host immunity against the vector backbone. The China group published their first set of clinical data on 15 August 2020.^98^ In this phase 2 trial, the vaccine was found to induce significant immune responses in the majority of the recipients after a single immunization. No data from their phase 3 study are available at the time that this review was prepared.

The clinical study data on AZD1222 vaccine (formerly known as ChAdOx1) was published on 19 December 2020 and 9 January 2021.^111,112^ Overall, the vaccine has an acceptable safety profile, well tolerated in older adults, has similar immunogenicity across all ages, and effective against symptomatic COVID-19 in the interim analysis. However, an error in dosing where nearly 3000 participants were given half dose in their first vaccination and full dose in their second vaccination led to better protection at 90%, compared to those who received two full doses and had protection at 63% (overall at 70%). The reason why a lower dose produced a more robust immune response remains to be determined, but it is possible that the lower first dose may induce less host immune response to the vector so that the second dose may be more effective in inducing a better response. This vaccine has already been approved in the UK. The vaccine developer is now conducting a study on 30,000 subjects in the USA and may submit for FDA approval in the second quarter of 2021. Just before we submitted this review, this vaccine was reported to be less effective for the South African variant. For the South African trial of the vaccine, conducted in ~2000 people, the efficacy against the mild and moderate disease was reported to be ~22%, and this would not meet the minimum international standards for emergency use. Data are not yet peer-reviewed.

China reported the first rAd5-based COVID-19 vaccine on 29 February 2020, ~2 months after the viral genome was reported, in a news release with a video showing them receiving the vaccine (code name Ad5-nCoV). They reported their phase 1/2 data on 22 May 2020 (and paper published on 13 June 2020), showing that the vaccine was safe and a single dose was able to trigger both humoral and cellular immune response in a dose-response fashion.^113^ On the day before we submitted this review (8 February 2021), their phase 3 study in Pakistan was announced, and an overall protection efficiency of COVID-19 was 75% and the protection of severe/critical COVID-19 was 100%. They also claimed on 1 February 2021 that the overall response rate for their phase 3 study conducted in five countries with 40,000 people showed an overall protection efficiency of 66% and a protection against severe/critical COVID-19 to be 91%.^114^ Note that these response rates were generated with only one dose of the Ad-nCoV vaccine. There is no data yet on the effect of this vaccine against the South Africa SARS-CoV-2 variant.

The Russian heterologous COVID-19 vaccine (rAd26 and rAd5-based) was shown in a phase 1 trial to induce a good humoral and cellular immune response and was also found to be safe in a report published on 26 September 2020.^99^ On 2 February 2021, the phase 3 study interim data on 20,000 subjects were reported. From 21 days after the first dose of vaccine (the day of dose 2), 16 subjects (0.1%) from the vaccine group and 62 subjects (1.3%) from the placebo group were confirmed to have COVID-19, giving a vaccine efficacy of 92% in protection against COVID-19 disease.^115^ The vaccine was well tolerated. Note that the Russian health authority has already approved this vaccine with the phase 2 data on 11 August 2020.

The rAd.26.CoV.2.S/JNJ-78436725 vaccine has had their phase 3 data released in a press release on 29 January 2021. The vaccine, which requires only a single injection (as claimed by the company), can also be stored in a refrigerator for months. The interim analysis assessed 468 cases of symptomatic COVID-19 among 44,325 adult volunteers and the investigational vaccine was 66% effective at preventing the study’s combined endpoints of moderate and severe COVID-19 at 28 days post vaccination among all volunteers, including those infected with an emerging viral variant. They also claimed that it is 85% effective in preventing severe COVID-19. FDA recently announced that there will be an advisory meeting to discuss the request for EUA for their vaccine on 26 February 2021. Recently, the company also reported that the protection against mild disease in South African was weaker, at 57%.

### Inactivated virus vaccine

For inactivated vaccines based on the more traditional vaccine manufacturing methods, results of a phase 1/2 trial on BBIBP-CorV were published on 1 January 2021, demonstrating a good safety profile and the identification of the dose for phase 3 development. On 31 December 2020, the Chinese health authority announced that this vaccine was granted conditional marketing authorization based on the interim analysis of its phase 3 trial, which showed 79% efficacy against COVID-19.^116^ Another company was developing a COVID vaccine based on a similar approach (code name CoronaVac) and published their phase 1/2 clinical trial on 3 February 2021; the vaccine was found to be safe, but the selection of a dose was needed for confirmatory studies.^117^ A third institution is also developing an inactivated COVID-19 vaccine.^101^ Again, in a publication on 13 August 2020 on the data of their phase 1/2 studies, they had shown that their inactivated vaccine was safe and immunogenic. Phase 3 data for all these vaccines are not yet available.

There are also other companies in different countries trying to develop inactivated vaccines and to combine this with different adjuvants that may further boost its efficacy.

### Recombinant viral protein vaccines

Another approach is to use recombinant viral protein as a vaccine. One of the companies is using recombinant spike protein nanoparticle vaccine (code name NVX-CoV2373, which composed of a trimeric full-length spike glycoprotein and their Matrix-M1 adjuvant). On 2 September 2020, the phase 1/2 trial data were published.^118^ The data showed that this vaccine appeared to be safe, and it elicited an immune response that exceeded levels in COVID-19 convalescent serum. On 28 January 2021, the company announced that their vaccine demonstrated nearly 90% efficacy against COVID-19, in a cohort where half the cases were due to the new UK variant. However, in a trial in South Africa, the overall vaccine efficacy was 49% largely due to the South African variant. In addition, findings from that study suggested that prior infection with the wild-type strain may not fully protect against new infection from the variant strain. The company announced that regulators from the USA, UK, Canada, and Europe had begun to review these data in a rolling submission process.

Also, along the line of using recombinant viral glycoprotein, Yang et al.^119^ described that a vaccine targeting the RBD of the spike protein can induce protective immunity. What was important is this study was the observation that the immunogenicity of the RBD domain is stronger than the entire spike protein. Also, this study defined the importance of vaccine adjuvant in the potential use of RBD glycoprotein in eliciting a better host immune response in animals. Phase 1 and 2 studies are currently ongoing (ChiCTR20000037518 and ChiCTR20000039994).

### Forward looking

It is important to note the following forward-looking points. First, similar to the mRNA vaccines, it is likely that the other vaccines are also effective in protecting against the COVID-19 disease but not the infection, and if this is the case, vaccinees can still be infected and help to spread the virus around. Use of facemask and social distancing will still be required to support the world to control this pandemic. Second, how long will immunity last after the vaccination? Do we need booster doses from time to time? The vaccinees will need to be followed up to address this question. The same question can be related to COVID-19-recovered patients: Do COVID-19-recovered patients need to receive COVID-19 vaccine? The CDC recommended that, based on the rapid reduction in serum-neutralizing antibodies in some recovered patients, they are recommending that COVID-19 patients should also receive COVID-19 vaccine.^120^ Third, SARS-CoV-2 is an RNA virus and this type of virus can easily mutate to “escape” the immune pressure. With regards to the immune selection, the current data suggest that the South African variant will represent a major challenge to the vaccine development for universal coverage of all SARS-CoV-2 variants. Even when new vaccine candidates are developed to tackle the South African viral variant, it is possible that there may be newer variant evolving and be revealed with additional immune pressure. We can expect an array of data in this direction in the next couple of years. A recent publication by Kemp et al.^121^ also revealed the strong selection on SARS-CoV-2 during convalescent plasma therapy associated with the emergence of viral variants with the evidence of reduced susceptibility to neutralizing antibodies. The understanding of the interplay between the humoral response and the viral adaptation will be important for us to design a better vaccine and immunotherapy for COVID-19.

## Conclusions

The COVID-19 pandemic has challenged the world not just in the global health but also the global psychosocial and economic health. This pandemic is testing our resolve to solve challenging situation together. The scientific world has taken on this challenge and is investigating this virus, the COVID-19 disease, and pathogenesis, and have developed systems in epidemiology, diagnosis, clinical management, and development of vaccines in a timeline that is unprecedented (all within 1 year). This brief summary tried to describe some of the development and also the unanswered questions, with an attempt to use this information to allow us to look forward. To all the unsung heroes who worked so far to help the world combat this pandemic, we would like to share this quote from Sir Isaac Newton:

If I have seen further, it is by standing upon the shoulders of giants.
